# Knowledge and Practices on the Prevention and Management of Diarrhea in Children Under-2 Years Among Women Dwelling in Urban Slums of Karachi, Pakistan

**DOI:** 10.1007/s10995-022-03391-9

**Published:** 2022-03-05

**Authors:** Asif Khaliq, Amreen, Nazia Jameel, Stefanie J. Krauth

**Affiliations:** 1 Department of Community Medicines, Baqai Medical University, Karachi, Pakistan; 2 School of Public Health and Social Work, Queensland University of Technology, Brisbane, Australia; 3 Department of Paediatrics and Child Health, Aga Khan University Hospital, Karachi, Pakistan; 4 Department of Psychiatry, Dow University of Health Sciences, Karachi, Pakistan; 5 Institute of Biodiversity, Animal Health and Comparative Medicine, University of Glasgow, Glasgow, UK

**Keywords:** Paediatric diarrhea, Diarrhea management, Diarrhea prevention, Children, Knowledge and practices, Urban slums, Pakistan, Breastfeeding

## Abstract

**Background:**

Diarrhea is the second leading cause of death especially among children. The age-proportionate mortality of diarrheal disease in infants under 2 years is 72%, among children under 5 years of age. Children living in urban slums are more prone to develop diarrhea. Although the disease can be prevented by many simple cost-effective interventions, i.e. proper sanitation and hygiene, appropriate feeding, and timely vaccination, poverty and lack of basic life amenities often potentiate diarrhea mortality. Gadap town is the largest town of Karachi with a deprived health system. This study aims to assess pediatric diarrhea prevalence and related knowledge-practice gaps in the slums of Gadap Town, Karachi, Pakistan.

**Method:**

A community-based cross-sectional study was conducted from November 2016 to May 2017 among mothers of children under 2 years, who were residents of Gadap Town, Karachi, Pakistan. The participants were approached by a multistage sampling method. A validated dichotomous questionnaire, piloted on 40 participants, translated into local language Urdu was used for data collection and the data was analyzed by SPSS® version 20.0.

**Results:**

51.8% (n = 199) of participants were aged between 25 and 34 years. Among all participants, 68% (n = 261) had primary level education or less, compared to 4.7% (n = 18) of women who had graduate-level education. The mean number of children per woman was 2.52 ± 1.62. Self-reported pediatric diarrhea incidence was 72.1% (n = 277). More than half (55.2% n = 149) of participants reported frequent diarrhea episodes during the 2nd year of their child’s life. In this survey, we found the knowledge of women regarding diarrhea management and how to reduce diarrhea morbidity to be inadequate (p > 0.05). However, many women reported appropriate practices which can significantly reduce diarrhea morbidity (p < 0.05).

**Conclusion:**

While the knowledge among women on preventive measures for pediatric diarrhea was insufficient, the translation of the right knowledge into appropriate practices showed promising outcomes for reducing diarrhea morbidity. An integrated approach for improving feeding, sanitation, and hygiene practices along with continuous health education could curtail the burden of diarrhea among infants living in urban slums.

## Significance

*What is already known in this subject?* The knowledge and attitude of caregivers is important in the prevention and management of diarrheal disease in children under 5, but the translation of this knowledge into practices, especially in resource-limited settings, is often lacking for various reasons.

*What our study adds*: We confirm that knowledge on diarrhoea prevention does not adequately translate into practices in the slum area of Gadap town, Pakistan, partly also due to limited access to services and goods. However, adequate diarrhoea management practices, especially around feeding, were often reported even in the absence of formal knowledge. Nonetheless, some inadequate practices, such as caloric restriction, still prevail in the community.

## Introduction

Diarrhea is the 2nd leading cause of death in children under 5 worldwide, with an estimated 71.59 disability-adjusted life years (DALY) and 1.3 million deaths in all age groups (Walker et al., 2013; World Health Organization, 2017). Diarrhea can be defined as three or more unformed stools in 24 h and can lead to severe fluid loss, and electrolytes disturbances (Boschi-Pinto et al., 2009; World Health Organization, 2017). Children aged less than 5 years are disproportionally affected by diarrheal diseases, and the proportion of death attributed to diarrhea among children of this age group rises to 15 (World Health Organization, 2018).

The incidence and the mortality rate of diarrhea are high during infancy because children start crawling, walking, and generally interacting with their environment at this age (Gupta et al., 2015). Additionally, caretakers start supplementary feeding and weaning practices and weaned children lose some of the protection that is provided through breast milk while their immune system still has to build resilience against pathogens (Morrow & Rangel, 2004; Piechulek et al., 2003). The burden of communicable diseases is high among urban slums and *goths* (slum-like small neighborhoods) (Sarkar et al., 2013). Children living in urban slums are more prone to developing diarrhea compared to children living in other areas of the same city because they often live without basic amenities, infrastructure, and access to health and community services (Fink et al., 2014). In slums, the risk of diarrhea mortality is 2.5 times higher, especially where women are not autonomous in taking healthcare-seeking decisions (Agarwal & Taneja, 2005; D’SOUZA, 2003). However, diarrhea incidences can be reduced through simple public health interventions such as increased hygiene practices, proper sanitation, clean water supply, exclusive breastfeeding until the age of 6 months, and continued breastfeeding through 24 months (Black et al., 2003; Turin & Ochoa, 2014).

According to the World Health Organization’s (WHO) Global Health Observatory data for 2017, Pakistan currently ranks 23 (out of 194 monitored countries) for childhood (0–4 years of age) mortality due to diarrheal diseases. As such, diarrheal diseases accounted for 6 deaths in every 1000 live births in Pakistan in 2017 (World Health Organization, 2019). Karachi, situated in the Sindh province, is Pakistan’s most populated city with 14.9 million inhabitants (Pakistan Bureau of Statistics, 2017a). Gadap town, in the northwestern district of Karachi, is the largest town of Karachi and has known issues around the utilization of healthcare services and care-seeking behavior of the population which need to be addressed (Aleemi et al., 2018). Several studies have assessed diarrhea rates and associated risk factors in children under 5 years in Karachi. However, information on the knowledge and practices of children’s caregivers is still lacking for the area.

The aim of this study was to assess the current knowledge and practices of mothers of children < 2 years of age in Gadap Town, Karachi, Pakistan. This study provides the first local evidence about reported diarrhea prevalence among children under 2 years linked with information on knowledge and practices about diarrhea management among women dwelling in slum-like neighborhoods in several union councils of Gadap Town, Pakistan.

## Methods

### Description of Study Site

Gadap Town is the biggest town of Karachi, situated in a Northwestern part of Karachi with a population of around 64,192 (Pakistan Bureau of Statistics, 2017b). Most areas of Gadap town have a rural infrastructure. The town is divided into 8 union councils (UC): UC-01-Murad Memon, UC-02-Darsano Channo, UC-03-Gadap, UC-04-Gujro, UC-05-Songal, UC-06-Maymarabad, UC-07-Yousuf Goth, and UC-08-Manghopir. All UCs of Gadap towns exhibit similar sociodemographic and socioeconomic profiles. The health and educational infrastructure of Gadap town is limited with two state colleges and two maternity hospitals in the whole of Gadap town. Moreover, issues related to poverty and illiteracy are a common sociodemographic feature in all UCs of Gadap town leading to limited access to basic services (DAWN, 2005).

### Study Design, and Sampling

**Fig. 1:**
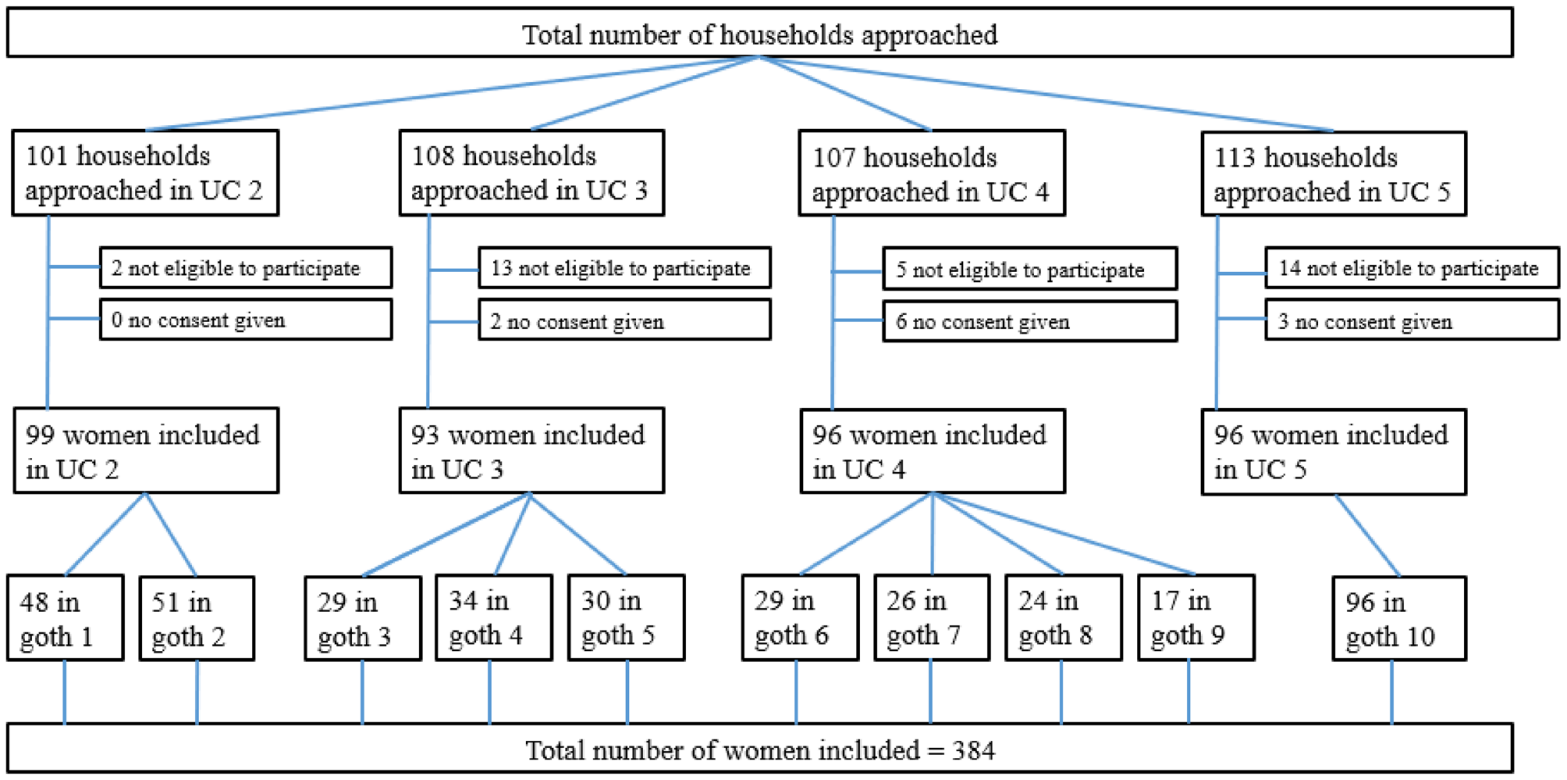
Participants selection in the study

In this study, a multistage sampling technique was used. In a first step, four out of 8 UCs of Gadap town were selected randomly by drawing paper lots. Within each selected UC, 1–4 goths (slum-like small neighborhoods) were chosen using a convenience sampling method under the guidance of local authorities and based on security concerns, feasibility, and permission by the local neighborhood authorities (Fig. 1).

The survey was performed in collaboration with Lady Health Visitors (LHV) and Community Health Workers (CHW) of the Baqai Medical University. Data was collected in several goths of the selected UCs in Gadap Town by the first author of this manuscript.

### Study Population and Eligibility Criteria

The study was focused on women with children under the age of 2 years due to the high age-specific proportional mortality rate for diarrheal diseases (72%) in the first 2 years of life (Walker et al., 2013). All women who have lived in one of the selected goths of Gadap Town for at least 2 years before the survey, who were able to understand and speak the Urdu language and had at least one child below the age of 2 years at the time of the study, were eligible to participate in the study provided they gave their informed consent to participate. If a woman reported having more than one child aged below 2 years, information regarding the diarrheal episodes and diarrhea management was collected for the youngest child. Women with twin or triplet children were excluded from the survey, because twin or triplet children are more prone to develop various growth and developmental disorders than singleton children (Shinwell et al., 2009). We excluded caregivers other than mothers because one of the main practices of interest revolved around breastfeeding practices during diarrheal episodes and in general. Moreover, parenting practices can vary greatly depending on the exact relationship between children and caregivers (Bornstein et al., 2016). Guests and visitors dwelling in the household at the time of data collection, as well as women who refused consent, were excluded from the study.

### Sample Size and Sampling Technique

Using the formula S = Z^2^ × P × Q / e^2^ (with Z^2^ = 3.84; P = 50%; and Q = 50%), the final sample size was calculated at 384, keeping population distribution of 50% and a confidence of 95%. The calculated sample size was verified using the Raosoft sample size calculator (Raosoft, 2004). The total sample size for the study was divided into 4 equal quarters leading to 96 individuals to be sampled within each UC.

No registries of the population in the different UCs of Gadap Town with up-to-date age-specific demographic statistics were available and no exact information regarding children under 2 years dwelling in Gadap Town was obtainable. Hence, random sampling of eligible households within the selected goths was not possible. We therefore employed a spatial sampling approach adapted from the UN Extended Programme of Immunization (EPI) (Bennett et al., 1994). In short, we intentionally chose an area within each goth where we encountered many houses and people. Subsequently, a walking direction was decided semi-randomly using the following approach: a direction was selected randomly by turning a pen. The resulting direction was then assessed for safety and accessibility (number of stray dogs, obstacles blocking access in the direction, etc.). If the direction was deemed unsafe by the field agent’s assessment, a new direction was selected using the same method. Continuing in the selected direction, every third dwelling was approached and inquiries were made about the presence of eligible individuals. In case no eligible individuals were present in the selected household at the time of our visit, the next dwelling closest to the originally selected household in the same walking direction was approached instead. Data collection was performed from December-2016 to May-2017 between 09 and 13 h.

### Ethical Considerations

Ethical clearance was obtained from the Advisory Committee of the Baqai Institute of Health Sciences; Baqai Medical University, Pakistan. The committee approved the conduction of the household survey in Gadap Town, Karachi, Pakistan. Data was collected from eligible and consenting participants observing all ethical principles of beneficence, non-malevolence, justice, and equity. The purpose of the study as well as associated risks and benefits thereof were explained to all eligible individuals and a written information and consent form was provided to all eligible individuals. The women were then given adequate time to ask any questions and consider their participation. All illiterate participants received a detailed oral explanation of the study and were asked for oral consent as well as a thumb impression on the consent form in the presence of other community members of their choice. Information regarding data anonymization and confidentiality was also provided to all participants during the informed consent procedure. Verbal and written informed consent were given by all participants before the commencement of the questionnaire survey and all participants were explicitly informed about their rights to refuse participation or withdraw from participation at any point in time without any negative consequences.

## Measures

A questionnaire was designed based on a thorough literature review on important knowledge about diarrhea in children and on suitable prevention practices. The questionnaire was drafted in English and then translated into the local language Urdu by a bilingual member of the research team. The questionnaire content and face validity were checked by 2 public health specialists and one epidemiologist of Baqai Medical University and then pilot tested. The pilot testing was performed by administering the questionnaire to 40 participants (8–13 participants from each UCs). The data of the pilot test was not included in the final analysis. The Cronbach alpha value after pilot testing was 0.679 and this value is near to the minimum acceptable limit (0.7 or more) of Cronbach alpha for reliability analysis (Taber, 2018). After pilot testing, the study design was approved by the Baqai Institute of Health Sciences, at the Baqai Medical University, Karachi, Pakistan.

The final questionnaire consisted of the following sections: Demographic and family information, diarrhea medical record, and knowledge & practice assessment. The demographic section asked for information about the participant’s age, education, and the number of children present (excluding children of any other family member). The diarrhea medical record section contained questions about the diarrhea history of the children below 2 years of age and the age at which the women believed that their child had frequent and severe episodes of diarrhea during the last 12 months.

The knowledge and practice section contained a total of 20 questions about (a) sanitary and hygienic measures (water boiling, water filtration, chemical disinfection, hand washing), (b) dietary measures (exclusive breastfeeding, oral rehydration solution (ORS) use, feeding bananas, feeding yogurt, and food abstinence) and (c) public health preventive measures (vaccination). For each answer that concurred with currently recommended behaviors in diarrheal management, the respondents were given 1 point, counting towards a simple summary score. For each answer that did not concur with current recommendations, the respondents got zero points. This way, a participant could obtain a maximum of 20 points if they scored 10 points in the knowledge section and 10 points in the practice section.

The responses of each participant were categorized as sufficient or insufficient knowledge and as suitable or unsuitable practices, based on the cumulative responses in the respective section. A score of 70% or more in either section was considered to show: *“sufficient knowledge”* and *“suitable practices”*, respectively*.* A score of less than 70% was categorized as: *“insufficient knowledge”* and *“unsuitable practices”*, respectively.

### Data Quality Management and Statistical Analysis

The data of this research was double entered by a single person into Microsoft Excel 2010 and the Statistical Package for Social Sciences (SPSS) version 20.0 and checked for inconsistencies. If discrepancies were found, they were corrected referring to the paper data collection forms. All data collected were kept under the custody of the principal investigator. All data was entered without personal identifiers such as names or locations (goths).

Descriptive statistics of quantitative data were analyzed using SPSS and Microsoft Excel. The normal distribution of the data was checked with the Kolmogorov–Smirnov test which produced a p-value of < 0.001. With a p-value less than α < 0.05, we concluded that the data of our study was not normally distributed and therefore used non-parametric tests for the inferential analysis of data. The association of diarrhea episodes with the participant’s knowledge and practices was measured by calculating the odds ratio.

To understand the association between diarrhea episodes and predictor variables such as the participant’s age, education, number of children in total, number of children under 2 years, age of the children, and the total score of woman’s knowledge and practices around diarrhea, multiple linear regression was applied in SPSS.

## Results

The following UCs were selected for the study: UC-02-Darsano Channo, UC-03-Gadap, UC-04-Gujro, and UC-05-Songal.

- **UC-02-Darsano Channo:** 1-Dunmba goth, 2-Zakariya goth
- **UC-03-Gadap:** 3-Konkar, 4-Haji Ismail goth, 5-Faqeer Sohrab goth
- **UC-04-Gujro:** 6-Faqeera goth, 7-Lassi goth, 8-Ayub goth, 9-Yasrub colony
- **UC-05-Songal:** 10-Ghulam Muhammad Goth.

### Participants’ Demographics

A total of 429 houses were approached during this study, out of which 34 did not fulfill the eligibility criteria, and 11 households refused participation. Detailed numbers of included women per Goth can be seen in Fig. 1.

In total, 384 women with children under 2 years of age participated in the survey. The mean age of the participants in our survey was 28.4 (± 6.3) years, ranging from 18 to 48 years with the majority of women (51.8%) aged between 25 and 34 years, followed by 27.8% aged between 15 to 24 years. A total of 261 (67.9%) women were illiterate or had no education beyond primary level education, whereas 18 (4.7%) women reported having a graduate-level qualification. The mean number of children present in each household was 2.52 ± 1.62, ranging from 1 to 9 children. The mean number of children aged less than 2 years was 1.30 ± 0.51, ranging from 1 to 3 and there were 106 (27.6%) households where more than 1 child under the age of 2 years was present at the time of data collection.

### Diarrhea Episodes and Child Age

In this study, 72.1% (n = 277) of women responded that their children suffered from 3 episodes of diarrhea in the last few months. A total of 149 (53.8%) women who reported episodes of diarrhea for their children, reported that their children had the most frequent and severe episodes of diarrhea around 12–23 months of age (Table 1).

**Table 1:** Pediatric diarrhea incidence and age

Variables	Categories	Frequency (n)	Percentage (%)
Self-reported diarrhea incidence in Children in the last 12 months	Yes	277	72.1%
	No	107	27.9%
Child age at diarrhea episode* (n = 270)	Under 1 year (0–11 months)	121	44.8%
	Under 2 years (12–23 months)	149	55.2%

*Seven women didn’t know the age of their children at the time of diarrhea incidence

### Water Source and Water Taste

The most common source for water supply was reported as bore/well-water by 230 (59.9%) women, followed by tap-water from 66 (17.2%), tanker supply from 48 (12.5%), and hand-pumps by 32 (8.3%) women. Eight (2.1%) women complained that the water supply is generally not appropriate. Less than half of the participants (n = 175, 45.6%) reported having access to fresh-tasting drinking water with 209 (54.4%) women stating that the taste of their drinking water was either saline or brackish.

### Reported Diarrhea Management Knowledge and Practices

The knowledge of women on sanitary measures to prevent diarrhea such as treating water before consumption and handwashing was sufficient with more than 80% of women stating that hand washing, and boiling water could help prevent diarrhea. Knowledge of other suitable measures to treat water was insufficient (Table 2). The level of reported preventive practices lacked behind the knowledge on these measures by 20–40%, indicating that women do not always implement their knowledge. The exception was handwashing practices, where 84.9% of women agreed that handwashing can prevent diarrhea and 83.1% also reported washing their hands before preparing food. Moreover, the reported knowledge and practices of women regarding the use of oral rehydration solution (ORS) and feeding bananas in the management of diarrhea was more than 70%, indicating that women have sufficient knowledge on feeding practices during diarrheal episodes and follow appropriate practices. The level of sufficient self-reported feeding practices during diarrheal episodes was generally high and, overall, even slightly higher than the knowledge around these practices (64.7% appropriate knowledge vs 68.8% appropriate reported practices), indicating that women performed some adequate diarrhea management practices even in the absence of formal knowledge. In total, 67.7% (n = 260) of women continued breastfeeding during diarrheal episodes (knowledge on the benefits of breastfeeding was shown by 65.5%; n = 244), and 80.2% (n = 302) reported using ORS (knowledge: 88.5%; n = 340) while 88.5% (n = 340) reported to feed bananas during their children’s diarrheal episodes (knowledge: 74.6%; n = 286). Although 78.6% (n = 302) of women believe that vaccinations can prevent episodes of diarrhea in young children, only 2.9% (n = 11) reported having vaccinated their children against pathogens causing diarrhea (Table 2).

**Table 2:** Women’s knowledge and practices regarding diarrhea preventive and promotive measures

Measure	Knowledge	Practices
	% (n)	% (n)
Sanitary and hygiene preventive measures		
Water boiling	86.5% (n = 332)^∞^	56.3% (n = 216)
Water filtration	56.8% (n = 218)	22.4% (n = 86)
Water chemical disinfection	46.4% (n = 178)	22.7% (n = 87)
Hand washing	84.9% (n = 326)^∞^	83.1% (n = 319)^¥^
Dietary measures		
Exclusive breast feeding	63.5% (n = 244)	67.7% (n = 260)
ORS use in diarrhea management	88.5% (n = 340)^∞^	80.2% (n = 308)^¥^
Banana use in diarrhea management	74.5% (n = 286)^∞^	88.5% (n = 340)^¥^
Yogurt use in diarrhea management	74.2% (n = 285)^∞^	67.7% (n = 260)
Not restricting food and water intake during diarrhea*	20.6% (n = 79)	39.8% (n = 153)
Mass preventive measure		
Vaccination and diarrhea	78.6% (n = 302)^∞^	2.9% (n = 11)

*Indicate negative scoring, i.e. all types of food restriction during diarrhea is not a healthy practice, ∞ indicates adequate knowledge of women regarding diarrhea preventive and therapeutic measures, ¥ indicates appropriate practices of women regarding diarrhea prevention and management

Despite overall adequate knowledge and practices on diarrhea management by the women in this study, 60.1% (n = 231) of participants responded that they cease to give any food or water for at least some time during a diarrhea episode of the children (Table 2) which constitutes an outdated and potentially harmful practice.

### Association of Knowledge and Practices with Diarrhea Episodes

Sufficient knowledge was not significantly associated with a reduction the odds of (self-reported) diarrhea among children (state OR, p > 0.05), whereas adequate practices for diarrhea prevention and management significantly reduced diarrhea episodes among children reduced the odds of diarrhea 2.62 times (p = 0.017) (Table 3).

**Table 3:** Pediatric diarrhea association with women’s knowledge and practices

Knowledge & practice measures	Diarrhea prevalence in children			p value	Odds ratio	Confidence interval
	Yes	No				
Women’s knowledge regarding pediatric diarrhea preventive measures						
Adequate knowledge	160		116	0.353	0.891	0.565–1.406
Inadequate knowledge	65		42			
Women’s practices regarding pediatric diarrhea preventive measures						
Appropriate practices	262		15	0.017	2.629	1.223–5.655
Inappropriate practices	93		14			

### Predictors for Effective Pediatric Diarrhea Management Practices Among Women

A multiple linear regression model was significant for diarrhea management practices among women, F (8, 375) = 6.510, p < 0.001, R^2^ = 0.122. The model shows that 1% of the variance is accounted for by the predictor variables. Factors that predicted adequate diarrhea management practices among women include having more than one child under 2 years of age (B = 0.156, p < 0.002), and adequate knowledge score about diarrhea case management practices (B = 0.228, p < 0.000). These findings suggest that an increased number of children under the age of 2 and increased knowledge about diarrhea case management are associated with better self-reported management practices of diarrhea (Table 4).

**Table 4:** Coefficients of multiple linear regression predicting practices about diarrhea management among women

Variable	B^a^ ± SE	Βeta^b^	p value	95% for CI for B	
				Lower	Upper
Age of participant	− .032** ± **.054	− .130	.556	− .138	.074
Education of participant	− .018** ± **.041	− .022	.658	− .100	.063
Number of children	− .020** ± **.053	− .020	.710	− .123	.084
Number of children under 2 years	− .484** ± **.156	.156	.002*	− .791	− .177
Knowledge total	.166** ± **.036	.228	.001**	.095	.237
Age of children	.210** ± **.267	.173	.432	− .315	.736

^a^Unstandardized sample regression coefficient

^b^Standardized sample regression coefficient

adjusted R^2^ = 1.22, overall model F test = 6.510, p = .000

^*^p < .005

^**^p < 0.001

## Discussion

Our study identifies an important gap in the knowledge and practices of women regarding preventive measures and the management of pediatric diarrhea. Overall, the knowledge of participants about prevention and management practices for pediatric diarrhea was generally higher than the self-reported applied practices for almost all assessed components (Table 2). The knowledge score was not significantly associated with a reduction in the incidence of self-reported diarrhea among participant’s children (Table 3). However, the self-reported application of appropriate prevention practices was associated with a reduction in self-reported diarrheal incidence in children below 2 years of age (Table 4). Appropriate practices reduced the odds of self-reported diarrhea by 2.62. This implies that participant’s practices are of importance together with adequate knowledge (Table 3). The findings of our study are consistent with other comparable studies (Kundu, 2010; Rumbo et al., 2016). Apart from a knowledge-practice gap, other factors which influenced women’s practices around diarrhea case management were examined. In general, an increased number of children under 2 years of age and an increased knowledge score were significantly associated with a higher score of women’s practices for diarrhea case management, although it has been previously shown that a higher number of children can increase the odds for diarrhea (Caruso et al., 2010).

Various studies that assessed knowledge and practices around diarrhea in children suggest that women may not be adequately aware of all important factors which make children vulnerable to diarrhea (Kundu, 2010; Rumbo et al., 2016). Our findings suggest that women have adequate knowledge regarding diarrhea case management but that this knowledge doesn’t always translate into appropriate practices which are significantly associated with the incidence of pediatric diarrhea. Translation of knowledge into practices could be an effective tool for controlling the disease proliferation in a population and should be a key public health strategy (Forsberg et al., 2007). The demographic characteristics of the present research participants indicated that 67.9% of participants were illiterate or had a primary level of education and only 4.7% of women reported having a graduate-level qualification. Table 4 further elaborates, however, that participants’ level of education was not a significant predictor of adequate diarrhea management practices in the present study, likely due to the small number of women with higher than primary education in our sample. Local research conducted by the lead author as well as other researchers in Pakistan indicated that less than secondary level education is one of the biggest risk factors for diarrhea prevalence among children under the age of 2 years (Arif & Naheed, 2012; Irfan et al., 2017). Another possible influence on women’s practices could be when women do not perceive their environment as supportive enough to adopt healthy lifestyle behaviors (Corcoran, 2013). A behavioral approach to health promotion focuses on the behavioral change process through active involvement in community and health promotion activities that enable people to adopt healthy behaviors in an appropriate way (Laverack, 2014). Such an approach to translate knowledge into practice in the slum areas of Pakistan could greatly help improve the case management of infant diarrhea among women in these areas.

This study showed that women in the slum areas of Gadap town had adequate knowledge on the use of Oral Rehydration Solution (ORS) for the management of pediatric diarrhea. Similarly, practices around the use of ORS were also appropriate. Although the ORS has no role in stopping diarrheal episodes, it is important for replenishing the electrolyte imbalance caused by fluid loss (Bham et al., 2015). These positive findings on the knowledge about, and use of, ORS during diarrheal episodes might be due to various health education programs implemented by the Government of Pakistan. The Lady Health Worker (LHW) program and education via mass media have enabled caregivers to identify and manage common preventable illnesses, including diarrhea (Malik et al., 2020; Ronis & Nishtar, 2007). However, several studies reported that mothers and other caregivers in Pakistan still treat diarrhea in children with home remedies or self-prescribed medication, including antibiotics, and only one third of caregivers manage pediatric diarrhea using ORS (Aftab et al., 2018; Khan et al., 2019). One of the main reasons for these findings is the inaccessibility and non-affordability of ORS which constitutes a major barrier for ORS therapy in Pakistan, especially in socioeconomically deprived areas (Ezezika et al., 2021). Thus, efforts should be made to increase the accessibility and affordability of ORS for example by providing subsidiary in ORS pricing.

One of the more concerning findings on the practices around diarrheal management concerns the reduction of fluid and food intake in young children with diarrhea. Restricting fluid and food intake during diarrheal episodes is a sometimes fatal practice because diarrhea itself is associated with fluid loss (Omoke et al., 2021). The practice of fluid and food restriction during diarrhea is very common in Karachi as well as in other parts of Pakistan. Aftab et al. (2018) showed that only one third of caregivers increase the fluid intake for their children during diarrheal episodes, while more than half of the caregivers generate a caloric deficit to their children by reducing the food quantity in the belief that this would stop diarrhea sooner (Aftab et al., 2018).

In Pakistan, a combination of a Kitchri (boiled rice and lentils mixture), Yogurt, and Banana diet is used by many caregivers during diarrheal episodes (Aftab et al., 2018; Bham et al., 2015). Many studies which were conducted in the past also showed a preference for this food for children with diarrhea (Bennett et al., 1994; Imran Ali et al., 2019; Zahid et al., 2014). However in our study, despite the participants’ awareness of the benefits of feeding yogurt and banana, the feeding of yogurt was not high. In contrast, the use of bananas far exceeded the women’s awareness of potential benefits which may be because bananas are one of the preferred foods when women start weaning their infants (Khaliq et al., 2017). Green banana and pectin can be used as a cost-effective and safe way the management of diarrhea (Rabbani et al., 2001).

Women who participated in this study believed that vaccination can be an effective tool for preventing diarrhea among children, but less than 5% responded that they have immunized their children with vaccines against infections causing diarrhea. Rotavirus vaccination was not part of the routine immunization program of Pakistan by early 2017 when this study was conducted (Alam et al., 2015). The low immunization rate with the rotavirus vaccine reported by women in this survey might therefore be due to the lack of rotavirus vaccine administration by the Government of Pakistan. Parents living in urban slums face important barriers to vaccinating their infants with vaccines that are not provided routinely because of high vaccine prices and low literacy (Apte et al., 2018).

Despite the high prevalence of pediatric diarrhea in children under 2 years of age, adequate knowledge, and appropriate practices of women about their handwashing before cooking and feeding as well as after defecation is shown. However, this study did not assess several other sanitation-related issues, such as type of water, quality and type of sanitation facilities, and excreta disposals practices (World Health Organization, 2006). Various studies reported a high prevalence of diarrhea in children of families with a low socio-economic status because of a reduced access to safe drinking water or proper toilets and sanitation facilities (Hubbard et al., 2020; Soboksa et al., 2021). The inaccessibility to safe drinking water refers to drinking water contamination with the fecal material, and the presence of fecal material in the drinking water reflects the presence of enteropathogens, which are responsible for various types of enteric diseases, including diarrhea, typhoid, cholera, and others (Loyola et al., 2020; Parvin et al., 2021). Similarly, the use of shared toilet facilities and unimproved toilet facilities also increased the odds of diarrheal and enteric diseases in children (Hubbard et al., 2020; Ishimwe et al., 2020). However, the use of soap after defecation has the potential to avert diarrhea due to environmental enteropathogens (Hubbard et al., 2020). The environmental factors related to sanitation and hygiene showed a profound effect on the high endemicity of diarrheal diseases. In this regard, efforts should be directed not only towards maternal socio-behavioral knowledge and practices but also towards the environmental factors of sanitation and hygiene.

## Limitations

The reported study was conducted based on self-reported episodes of diarrhea as well as self-reported knowledge and practices. As such it is subject to the well-known issues of recall bias and response biases inherent in interview-based and cross-sectional survey designs. No means of verification were used to validate the responses taken from women. The dichotomous nature of the questionnaire restricts the exploration of underlying knowledge and practices details of women. Many mediating and moderating variables, such as healthcare-seeking behavior, disease knowledge, dehydration assessment, dehydration management, use of antibiotics, micronutrients including zinc, and herbal products, seasonality, etc. was not discussed with the participants in this study. Moreover, the social desirability of certain answers to our questions will likely have influenced reported behaviors leading to underreporting of socially undesirable behaviors and overreporting of socially desirable ones. Nevertheless, a clear association between reported behaviors and diarrheal episodes in children could be seen, although the exact magnitude of this association might be skewed by participants giving answers, they deem desirable. An exploratory, qualitative-research study or a follow-up interventional trial would provide a more in-depth understanding of the relationship between knowledge and practices and diarrhea episodes. Such studies would provide a stronger evidence-based for the current practices and barriers that women face for the management and prevention of pediatric diarrhea.

## Conclusion

Diarrhea is a common childhood illness, which can be prevented by simple cost-effective interventions (Bham et al., 2015; Mohammed et al., 2018). Children living in slums are especially prone to develop various infectious diseases, including diarrhea. Although the findings of this study showed adequate knowledge regarding pediatric diarrhea management, the practices regarding appropriate preventive and management measures for pediatric diarrhea have been found to not be sufficient to overcome the disease morbidity. Efforts to translate existing knowledge into appropriate practices could help in the efforts towards reducing pediatric diarrhea morbidity. Practices around diarrhea case management could be improved by providing diarrhea case management training to mothers and caregivers according to the Integrated Management of Childhood Illness (IMCI) guidelines at institutional as well as at household and community levels. Together with health practitioners, other allied healthcare staff such as Lady Health Workers (LHW), and Community Health Workers (CHW) can educate and train mothers and caregivers regarding the prompt management of diarrhea case management. An integrated approach for improving feeding, sanitation, and hygiene practices along with continuous health education can curtail the burden of diarrhea among infants living in urban slums.

## Data Availability

The dataset generated for this study is under the custody of the primary researcher. The dataset will not be shared in an openly accessible database because the information to participants clearly stated that all data will be treated confidentially and will only be used for research purposes. Inquiries on the data should be addressed to the first author.

## Abbreviations

UC: Union council
DALY: Disability adjusted life years
WHO: World Health Organization
EPI: Expanded program for immunization
LHV: Lady health visitors
CHW: Community health workers
SPSS: Statistical package for social sciences
